# Target discrimination and PAM profiling of the *Thermotoga maritima* type I-B CRISPR system

**DOI:** 10.1042/BCJ20260189

**Published:** 2026-05-21

**Authors:** John Mallon, Charles J. Lenihan, Sowmyaa Shridhar, Scott Bailey

**Affiliations:** 1 Department of Biochemistry and Molecular Biology, Johns Hopkins University, Bloomberg School of Public Health, Baltimore, MD 21205, U.S.A.; 2 Department of Biophysics and Biophysical Chemistry, Johns Hopkins University, School of Medicine, Baltimore, MD 21205, U.S.A.

**Keywords:** CAS, Cascade, CRISPR, crRNA, PAM

## Abstract

Type I-B CRISPR–Cas (clustered regularly interspaced short palindromic repeats–CRISPR-associated proteins) systems represent the most abundant CRISPR subtype in nature and have emerged as powerful tools for endogenous genome editing in diverse prokaryotes. Here we reconstitute and characterize the type I-B1 system from the thermophile *Thermotoga maritima* (*Tma*) using purified components. We demonstrate that *Tma* Cascade requires standalone Cas11 expression, as the cryptic internal translation start site within *cas8b1* is non-functional in *Escherichia coli*. The reconstituted system exhibits canonical type I function including RNA-guided DNA binding, protospacer adjacent motif (PAM)-dependent target discrimination, Cas3-mediated degradation, and seed region interrogation spanning seven PAM-proximal nucleotides. Using next-generation sequencing-based PAM library screens, we define a YYD consensus PAM (Y = C/T; D = G/A/T) with strong discrimination against the array repeat-adjacent sequence (AAC). Comprehensive PAM profiling reveals context-dependent tolerance for non-consensus sequences and identifies numerous intermediate-activity PAMs that may function in priming. Comparison with other characterized type I-B systems reveals a correlation between the Cas8b variant and position −3 specificity, conserved pyrimidine preference at position −2, and variability at position −1. This work establishes a thermostable type I-B platform for biotechnological applications and provides insights into evolutionary mechanisms balancing PAM promiscuity with self-discrimination in the most abundant CRISPR–Cas subtype.

## Introduction

Clustered regularly interspaced short palindromic repeats (CRISPR) systems function as prokaryotic adaptive immune systems that defend against foreign genetic elements such as bacteriophages and plasmids [1,2]. A CRISPR array is a genomic locus consisting of short repetitive sequences (repeats) interspersed with unique variable sequences (spacers) derived from previous encounters with foreign genetic elements. These arrays are transcribed and processed into CRISPR RNAs (crRNAs) that serve as immune memory, with the spacer sequences guiding CRISPR-associated (Cas) proteins to recognize and destroy matching foreign nucleic acids [3]. The selective pressures imposed by these foreign elements have driven the widespread adoption of CRISPR systems among prokaryotes [4]. Similarly, the breadth of these selective pressures and of host cell diversity, combined with the horizontal mobility of CRISPR–Cas loci, has resulted in substantial diversification of these systems. Classification of CRISPR–Cas systems integrates multiple approaches, including phylogenetics, comparative genomics, and structural analysis. The current classification system defines 2 classes, 7 types, and over 30 subtypes [4]. Type I systems account for nearly 50% of all CRISPR–Cas systems, with type I-B being the most abundant.

In type I systems, invasive DNA is identified by an RNA-guided effector complex termed Cascade [3]. Cascade comprises multiple Cas proteins (Cas5, Cas6, Cas7, Cas8, and Cas11) assembled around a crRNA, with precise composition varying by subtype [5,6]. The crRNA contains a guide region derived from CRISPR spacers flanked on both ends by constant sequences derived from CRISPR repeats. Cas5 and Cas6 specifically recognize the 5′ and 3′ crRNA ends, respectively. Between these terminal proteins, several copies of Cas7 form a filament that positions the guide sequence in a solvent-exposed conformation competent for base pairing. The Cas8 and Cas11 subunits orient opposite to this Cas5–Cas7–Cas6 backbone and are both required for DNA binding.

Once Cascade binds foreign DNA matching its crRNA, it recruits the nuclease/helicase Cas3, which unwinds and degrades the target [7]. However, Cascade must employ a mechanism beyond crRNA complementarity to accurately identify foreign DNA. This is essential because the crRNA itself originates from the host’s CRISPR array, meaning that without additional discrimination criteria, the system would inadvertently target the host genome (self-targeting). Thus, target recognition also requires the presence of a PAM (protospacer adjacent motif), a short sequence motif located immediately adjacent to the complementary target sequence in the DNA [8]. The PAM is recognized by the Cas8 subunit of Cascade [9]. Evolutionary pressure to prevent self-targeting has driven PAM specificity to diverge significantly from the sequences bordering spacers within the CRISPR array [10,11]. An additional advantage of PAM-based screening is that it occurs while the DNA remains double-stranded, eliminating the need for extensive DNA unwinding. This allows Cascade to much more efficiently interrogate potential targets before committing to melting and pairing it to the crRNA [12].

Type I-B systems are not only the most abundant type I CRISPR system but also the most diverse [4]. Unlike other type I subtypes, their Cas8 subunits (Cas8b’s) do not form a monophyletic group due to this diversity. Instead, Cas8b proteins are classified into over a dozen variants (Cas8b1–Cas8b15) based on sequence similarity. Indeed, some Cas8b variants are more similar to Cas8 proteins from other subtypes (i.e., Cas8a or Cas8c) than to other Cas8b variants. Consequently, characterizing a single type I-B system provides limited insight into the target specificity of other I-B variants. Developing type I-B-based tools for diverse applications therefore requires broader characterization of how PAM specificities correlate with Cas8b variants across multiple systems.

Type I systems have diverse applications [13,14]. Type I-B being the most widely represented subtype is preoptimized in many prokaryotes, enabling endogenous editing and manipulation approaches. Hijacking endogenous type I-B systems has been exploited for efficient gene editing in prokaryotic cells [15–22]. In head-to-head studies, endogenous type I-B editors have outperformed benchmark *Streptococcus pyogene*s Cas9 while avoiding heterologous Cas9 cytotoxicity [15]. Endogenous gene knockdown methods (CRISPRi) have also been developed with type I-B systems [23–26]. Finally, bioinformatic studies have identified many naturally occurring type I effectors with novel protein associations or domain fusions, suggesting an array of evolutionarily optimized genome editors for further application [27,28]. For example, recently characterized transposon-coupled I-B Cascade systems from *Peltigera membranacea cyanobiont 210A, Rippkaea orientalis*, and *Anabaena variabilis* are natively capable of RNA-guided DNA transposition [29–31].

Here, we characterize the type I-B system from the hyperthermophile *Thermotoga maritima* (*Tma*), a system that contains a Cas8b1 gene. *Tma* has an 80°C growth temperature, a potential practical advantage, as thermostable CRISPR systems may exhibit enhanced stability in delivery vehicles such as virus-like particles and lipid nanoparticles [32]. We identify a necessary but cryptically encoded Cas11, as well as detailed insight into how this effector discriminates targets from nontargets with respect to PAM sequence preference and target mismatch tolerance.

## Results

### Reconstitution of *Tma* I-B system

**Figure 1:**
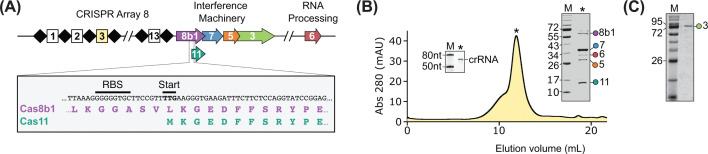
The type I-B interference machinery from *Tma* (**A**) Genome organization of the type I-B1 CRISPR system from *Tma*. The Cas8b1 (purple), Cas7 (blue), Cas5 (orange), Cas3 (green), and Cas6 (red) genes are indicated. Repeats (black diamonds) and spacers (squares) in CRISPR array 8 are also shown. The inset shows the internal translation start site of Cas11 (cyan) within the Cas8b1 gene. (**B**) Chromatogram of *Tma* Cascade purified by SEC. Right inset: SDS–PAGE of purified *Tma* Cascade. Left inset: denaturing PAGE of the crRNA co-purified with *Tma* Cascade. (**C**) SDS–PAGE of purified Cas3.

To assemble *Tma* Cascade, we co-expressed the crRNA with the Cascade subunits [33–39]. The crRNA was expressed from a synthetic CRISPR array containing seven identical spacer sequences, corresponding to the third spacer of the eighth CRISPR array from the *Tma* genome (which we call spacer 8.3 from here on out). For the Cascade subunits, we used genes encoding Cas7 (34 kDa) carrying an N-terminal His-SUMO tag, Cas5 (25.4 kDa), Cas6 (29.6 kDa), and Cas8b1 (62.7 kDa). We initially expected Cas11 (15.1 kDa) to be translated from the Cas8 transcript via an internal translation start site, as observed in other I-B, I-C, and I-D systems lacking a standalone Cas11 gene [40]. However, we did not observe Cas11 expression (Supplementary Figure S1), presumably due to a suboptimal ribosome binding site and/or start codon (Figure 1A). We also found that Cas8b1 did not purify with the rest of the complex in the absence of Cas11 (Supplementary Figure S1). As such, we added Cas11 to our co-expression experiments as a standalone gene. Following co-expression, affinity purification, and tag removal, the intact complex eluted from a size-exclusion column at a volume consistent with a molecular weight of ∼420 kDa. SDS–PAGE confirmed the presence of all Cas protein subunits, including Cas8b1 and Cas11 (Figure 1B). The complex also contained a crRNA that ran as a single band with mobility between the 50 and 80 nucleotide markers on a denaturing polyacrylamide gel (Figure 1B). This likely corresponds to the predicted mature 67-nucleotide crRNA, containing a spacer sequence flanked by repeat sequence at the 5′ and 3′ ends [41]. *Tma* Cas3 was cloned and expressed with an N-terminal His-SUMO tag, which was removed during purification (Figure 1C).

### RNA-guided DNA binding and cleavage by the *Tma* I-B system

**Figure 2:**
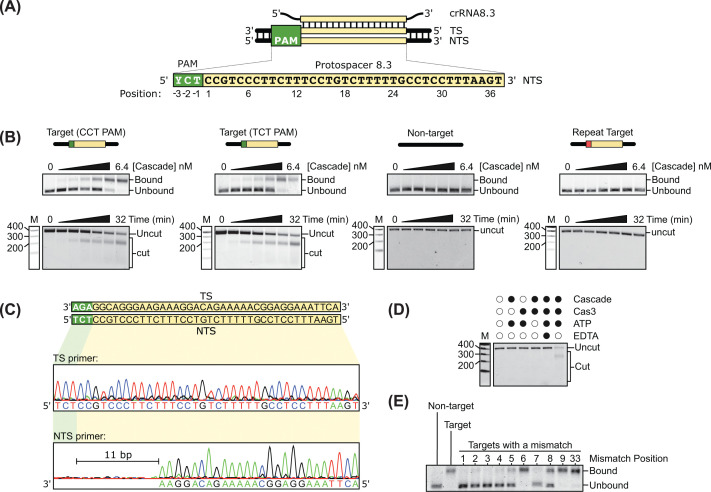
Reconstituted activity of the *Tma* type I-B CRISPR system (**A**) Schematic of the type I-B1 R-loop formed between the crRNA and DNA target. (**B**) Agarose gels showing (top) DNA binding and (bottom) DNA cleavage by Cas3 with target (CCT and TCT PAMs), non-target, and repeat target DNA. At the highest Cascade concentration, the intensity of the shifted band decreases relative to the preceding lane. Inspection of the full gels reveals a faint low molecular weight product (Supplementary Figure S2), suggesting that a small amount of nuclease contamination occurs under these conditions. (**C**) Sanger sequencing of the linear DNA cleavage product. (**D**) Agarose gel of DNA cleavage with components removed. (**E**) Agarose gel showing DNA binding to targets containing mismatches with the crRNA.

DNA binding and cleavage by type I systems require that the DNA target contain a protospacer flanked by a PAM [42]. Which sequences function as PAMs in the *Tma* type I-B system is not known but can be predicted bioinformatically due to an observed relationship between the sequence of the PAM and the CRISPR repeats, as well as by comparison to other closely related type I-B systems [42]. The bioinformatically predicted PAM for the *Tma* type I-B system is CCT (5′ to 3′ sequence on the non-target strand, the convention used here and on out), and TCT is a PAM used by three closely related type I-B systems [15–17] (Figure 2A). To determine whether these sequences function as PAMs and to establish that our reconstituted system is active, we compared the binding of *Tma* Cascade to a linear double-stranded DNA (388 bp) containing a protospacer flanked by each putative PAM (target DNA) and to a similar DNA lacking a protospacer (nontarget DNA). Increasing concentrations of *Tma* Cascade were incubated with each DNA at 80°C, the growth temperature of *Tma* [43]. Binding was then analyzed by agarose gel electrophoresis. With target DNAs we observed a progressive shift of the DNA band as the protein concentration increased (Figure 2B). In contrast, the non-target DNA band was not shifted (Figure 2B). At the highest Cascade concentrations, faint low molecular weight products were observed on the gels (Supplementary Figure S2). This likely reflects minor non-specific degradation under these conditions. This does not affect the interpretation of the binding data, as robust and specific gel shifts are observed across the lower concentration range used to assess PAM preference. PAMs not only facilitate target recognition but also provide a mechanism to avoid self-immunity [11]. CRISPR arrays, which contain protospacer sequences, lack PAMs and, as such, are not cleaved. AAC is the adjacent sequence found in the repeat of the *Tma* CRISPR array. We therefore tested binding to another target that contains a protospacer adjacent to the AAC repeat sequence (repeat target). Consistent with other CRISPR systems [42,44], this target was not bound by *Tma* Cascade (Figure 2B).

We next assessed the DNA cleavage activity of our reconstituted system. To do this, we preincubated the target, non-target, and repeat target DNAs with Cascade for 30 min at 80°C and then added Cas3 to initiate cleavage. At the indicated time points, samples of the reaction were taken, quenched with EDTA, and phenol extracted. The extent of cleavage was assessed by electrophoresis through agarose gels. Whereas the target DNA was degraded (Figure 2B and Supplementary Figure S2), no cleavage of the non-target DNA or repeat target was observed up to 32 min after addition of Cas3 (Figure 2B and Supplementary Figure S2). Target DNA cleavage resulted in a quasi-stable intermediate that runs between the 200 and 300 bp markers. To investigate this intermediate further, we gel extracted the band and Sanger sequenced it using primers that flank the protospacer region (Figure 2C). A clear interruption in the sequence of the non-target strand was observed 11 bases from the 3′-end of the PAM, suggesting that this is where Cas3 first nicks the target. In contrast, the sequence on the target strand was uninterrupted; thus, the intermediate is formed by the degradation of the non-target strand. Finally, cleavage of target DNA was not observed in control reactions lacking Cascade, Cas3, or ATP, or in reactions containing EDTA (Figure 2D).

CRISPR systems that degrade DNA, including type I systems, use a seed region within the protospacer, located directly adjacent to the PAM, to initiate base pairing with their targets [8,34,45]. Mismatches in the seed destabilize initial R-loop formation and therefore inhibit target binding and consequently degradation by Cas3 [8,34,45]. To define the seed region in the *Tma* type I-B system, we monitored binding to a series of targets containing sequence changes that introduce mismatches with crRNA8.3. Mismatches at positions 1 through 5 and position 7 each resulted in little or no binding. As expected, a mismatch at position 6 had no effect on binding, as every sixth base of the protospacer does not participate in base pairing [46]. A mismatch at position 8 had intermediate binding, while mismatches at positions 9 and 33 had comparable binding to a fully complementary target (Figure 2E). Collectively, these data suggest that the seed region for this protospacer sequence in the *Tma* type I system spans positions 1 though 5 and position 7, with position 8 as the boundary.

### The PAM profile of the *Tma* type I-B system

Diverse PAM sequences can elicit interference in type I systems [8,47–49]. Our reconstituted DNA binding and cleavage assays enable complete PAM profiling for this system, including weakly active PAM sequences that could represent low-frequency but potentially deleterious off-target sites. Type I-B PAMs comprise three base pairs. To determine which sequences function as PAMs in the *Tma* type I-B system, we generated a 3N library of dsDNA targets containing all 64 possible trinucleotide sequences adjacent to protospacer 8.3 and a non-target DNA negative control.

**Figure 3:**
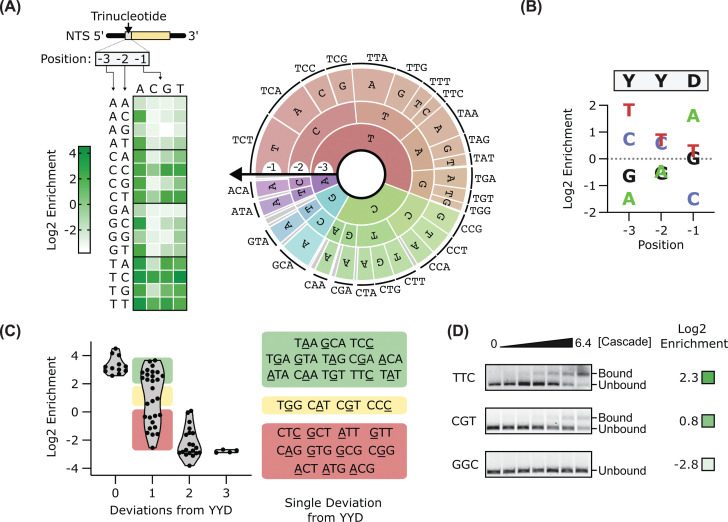
Effect of the PAM sequence on *Tma* Cascade DNA binding (**A**) DNA binding enrichment of each trinucleotide in the 3N PAM library is displayed (left) in a heatmap and (right) in a krona plot. (**B**) Plot of enrichments broken down per PAM position. (**C**) Violin plot of the enrichment data as a function of deviations from the YYD consensus sequence. (**D**) Agarose gels of DNA-binding assays performed with TTC, CGT, and GGC trinucleotide sequences. Full gels are shown in Supplementary Figure S3.

First, we determined which trinucleotides facilitate target binding by *Tma* Cascade. To this end, we incubated Cascade with the 3N library and separated bound and unbound library members by EMSA. The extracted bands were then PCR-amplified and subjected to NGS. From the NGS data, we calculated the enrichment in read abundance in the bound versus unbound pools for either each trinucleotide sequence (Figure 3A) or, separately, for each nucleotide at positions −3, −2, and −1, i.e., the positions that span the trinucleotide sequence (Figure 3B). In this screen, higher enrichment scores correspond to a higher degree of binding. Overall, this analysis shows that *Tma* Cascade recognizes a consensus YYD PAM (where Y is C or T and D is G, A, or T). This consensus includes the CCT and TCT PAMs. AAC, the trinucleotide sequence found in the repeat of the *Tma* CRISPR array, is one of the least enriched trinucleotides in our analysis, and at the level of individual nucleotides, A, A, and C are the least favored at positions −3, −2-, and −1, respectively (Figure 3A,B).

While our data identified a consensus YYD, several highly favored PAMs contain deviations from this motif (Figure 3A). To probe this further, we generated plots comparing the trinucleotide enrichments with an increasing number of sequence deviations from the consensus YYD (Figure 3C). While the YYD PAMs are all well bound (high enrichment scores), trinucleotides with 2 or 3 deviations from YYD either do not bind or bind poorly (low enrichment scores). Trinucleotides with one deviation display a range of outcomes. Those with similar enrichments to the YYD PAMs have either an A at position −1 or a T at position −3, both of which are the most favored base at their respective positions (Figure 3B).

To further validate the observed PAM preferences from the NGS activity data, we measured Cascade binding to an additional three single library members by EMSA. We chose these library members, which contained the trinucleotides GGC, CGT, and TTC, to sample a range of enrichment scores. The results from these single-target assays agree well with the NGS data, with measured single-target binding increasing with NGS enrichment score (Figures 2B and 3D and Supplementary Figure S3). Thus, the NGS data reflect the PAM profile of the *Tma* type I-B system.

**Figure 4:**
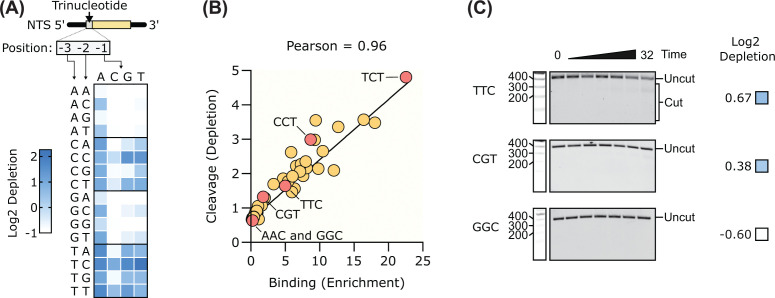
The Tma type I-B system can cleave targets containing a range of PAM sequences (**A**) DNA cleavage depletion of each trinucleotide in the 3N PAM library displayed in a heat map. (**B**) Plot of DNA binding enrichment versus DNA cleavage depletion. Trinucleotide sequences that were also tested individually in Figures 2B and 4C are highlighted. (**C**) Agarose gels of DNA cleavage assays performed with TTC, CGT, and GGC trinucleotide sequences. Full gels are shown in Supplementary Figure S3.

Next, we determined which targets can facilitate cutting by Cas3. To do this, we preincubated Cascade with the target library for 30 min and then initiated cleavage by the addition of Cas3. After 32 min, the reaction was quenched, and the uncut DNA gel was purified. The gel-purified starting library and the uncut fraction were then amplified by PCR and subjected to NGS. From the NGS data, we calculated the read abundance in the uncut and starting library pools. Targets that are cut more efficiently exhibit higher depletion in the uncut pool versus the starting library (Figure 4A). The heat map of these data (Figure 4A) is very similar to the heat map of the binding enrichments (Figure 3A). Indeed, a direct comparison of the depletion scores with the enrichment scores showed that Cas3 cleavage and Cascade binding are highly correlated under our assay conditions (Pearson’s *r* = 0.96) (Figure 4B). We also validated the cleavage data with a selection of single library members, which gave a range of depletion scores (trinucleotide sequences GGC, CGT, and TTC). The results of these experiments (Figure 4C and Supplementary Figure S3), as well as previous results for cleavage of targets containing CCT, TCT, and AAC trinucleotide sequences (Figure 2D), are consistent with the NGS depletion data.

## Discussion

Type I CRISPR systems are the most common in prokaryotic genomes and are increasingly being repurposed as efficient genomic tools, with I-B systems being the most commonplace and diverse subtype. Here, we reconstituted the type I-B1 system from the thermophilic bacterium *Tma* and determined its target recognition and cleavage properties. Our findings expand the characterized repertoire of type I-B systems and provide insights into PAM recognition sequence determinants, while establishing a thermostable system with potential biotechnological applications. The *Tma* system exhibits the hallmarks of canonical type I function: RNA-guided DNA recognition, PAM-dependent target discrimination, degradation by Cas3, and seed region-mediated target interrogation. The present work supports the consensus that type I systems share a common mechanism for target recognition and cleavage and that the rich diversity in type I systems is likely driven by selection pressure to avoid anti-CRISPR measures by phage [50].

Cas11 is encoded by many, but not all, type I systems. Type I-A and I-E systems encode a distinct Cas11 gene. Other type I systems lack a standalone Cas11 gene and either encode the small subunit C-terminally fused to the large subunit (types I-F and I-G) or have the small subunit generated by in-frame translation from the large subunit transcript (types I-B, I-C, and I-D) [40]. Although experimental demonstration in *Tma* is still required, it is highly likely that Cas11 is encoded within the Cas8b gene in type I-B systems, consistent with the in-frame translation mechanism described for related subtypes. When we overexpress *Tma* Cas8b1 in *E. coli*, we detect no small subunit, and therefore, to produce the intact *Tma* Cascade, we had to express Cas11 as a separate gene. This suggests that the internal translational start site for the *Tma* Cas11 gene is not functional in *Escherichia coli*. This observation has important implications for heterologous expression strategies. In addition, the inability to co-purify Cas8b1 in the absence of Cas11 suggests that Cas11 plays a critical structural role in complex assembly or stability and highlights the need to assess Cas11 expression when developing type I-based tools for use in exogenous systems, including other prokaryotes.

Our PAM library screens revealed that *Tma* Cascade recognizes a YYD consensus PAM while simultaneously disfavoring the repeat-adjacent sequence (AAC), demonstrating both the flexibility and evolutionary constraints that shape PAM recognition. We observed context-dependent tolerance in which some trinucleotide sequences containing single deviations from the YYD consensus display activity comparable to that of consensus PAMs. However, in these non-consensus PAMs the remaining two positions contained the most highly favored bases at their respective positions. These findings highlight the complex nature of PAM determinants and further demonstrate that consensus sequences alone do not fully capture the functional PAM space [49,51]. The anti-correlation between the repeat-adjacent sequence and PAM preference in *Tma* exemplifies the evolutionary pressure to avoid autoimmunity. Type I CRISPR–Cas systems discriminate against their own CRISPR arrays by excluding the repeat-adjacent motif from the set of functional PAMs [10,11]. This mechanism enables relatively promiscuous PAM recognition, which is important for a defense system aimed at fast-evolving phages, while maintaining self versus non-self discrimination. Collectively, these results underscore the complexity of PAM recognition and highlight the necessity of determining complete PAM profiles to fully understand the interplay between PAM promiscuity and self-discrimination in CRISPR–Cas systems.

Our PAM library screens identified numerous PAMs with intermediate activity that could represent priming PAMs (Figure 4B). Priming is an adaptive immune response in CRISPR–Cas systems whereby initial recognition of a mutated or suboptimal target triggers acquisition of new spacers from the foreign genetic element [52,53]. A key determinant of priming is PAM functionality. Targets bearing consensus PAMs typically undergo degradation, whereas PAM mutations or suboptimal PAMs can redirect the response toward priming. Many of the intermediate-activity PAMs identified in *Tma* differ from the consensus YYD PAM by a single nucleotide (Figure 3C), consistent with bona fide priming PAMs characterized in the *Haloarcula hispanica* type I-B1 system [54]. These observations are important because use of suboptimal PAMs in endogenous applications of type I-B systems may be complicated by unintended priming activity.

The full PAM profiles of 10 type I-B systems have now been characterized (Table 1). Comparing these PAM profiles reveals several broad trends. First, a correlation between Cas8b variant and position −3 (pyrimidine for Cas8b1 and Cas8b2, adenosine for Cas8b3) suggests that PAM recognition determinants have diverged among type I-B variants. Second, all characterized Cas8b’s recognize a pyrimidine at position −2, suggesting a conserved recognition mechanism at this position. And finally, PAM preferences are least conserved at position −1. Indeed, in several systems the PAM consensus at position −1 does not exclude the array repeat sequence^17, 54, 59, 60^ (Table 1). The PAM profile of the *Tma* type I-B1 *system* is most similar to that of the *Clostridium thermocellum* type I-B1 system [17]. Using a cell-based PAM depletion assay, the type I-B1 *C. thermocellum* system was found to use 10 PAMs, all of which are shared by the *Tma* system. Our characterization of the *Tma* I-B1 system using purified components provided evidence for a broader PAM profile, with around 25 trinucleotides acting as PAMs, albeit to varying extents. These differences likely reflect the methodology differences used to characterize the systems; similar trends were observed when comparing the PAM profiles of the *E. coli* type I-E system by different approaches [31,49].

**Table 1:** Type I-B systems with full PAM profiles.

Species	Cas8b	PAM	Repeat	Reference
*T. maritima* MSB8 0489932	1	YYD	AAC	The present study
*Hungateiclostridium thermocellum* DSM 1313	1	TTN/TNA	AAC	[17]
*H. hispanica* ATCC 33960	1	TTB/CCC	AGC	[54]
*Parageobacillus thermoglucosidasius* NCIMB 11955	1	TTA	AAC	[23]
*Pyrococcus* * furiosus* COM1	2	CCN	AAG	[59]
*Clostridium difficile* 630	2	YCN	AAT	[60]
*Synechocystis* sp. PCC 6714	3	AYG	CAC	[5]
*P. membranacea* cyanobiont 210A CAST	3	ATG	CAA	[29]
*Trichormus variablis* ATCC 29413 CAST	3	NAT	GCA	[29]
*R. orientalis* PCC 8801 CAST	3	ATG	CAA	[31]

N = A, T, G, or C.

Y = C or T.

D = G, A, or T.

B = C, G, or T.

A limitation of our study is that the PAM preferences determined using purified components have not been directly validated in cells. However, comparative studies in other type I-B and type I-E systems illustrate that PAM profiles determined in cells and with purified components generally agree for consensus PAMs but can diverge for intermediate-activity sequences [55,56]. Cell-based assays may miss weakly functional PAMs that are detectable biochemically. Conversely, cellular contexts capture the influence of factors such as DNA supercoiling, molecular crowding, and Cas3 recruitment dynamics that are absent from purified systems. Validating the *Tma* PAM profile in a relevant cellular context (in *Tma* itself or in a compatible heterologous host) therefore remains an important direction for future work and will be particularly valuable for assessing the activity of intermediate-activity PAMs and their potential contribution to priming or off-target effects in genome editing applications.

We have successfully reconstituted and characterized the type I-B CRISPR–Cas system from *Tma*, establishing it as a thermostable, RNA-guided DNA targeting platform with a well-defined YYD PAM consensus and a seed region spanning the PAM-proximal seven nucleotides of the protospacer. The requirement for standalone Cas11 expression highlights important considerations for heterologous reconstitution strategies. Our comprehensive PAM profile expands the characterized diversity within type I-B systems and reveals both conserved and variable features of PAM recognition across Cas8b families. The present work provides a foundation for the development of *Tma* I-B-based genome editing tools and contributes to our understanding of the evolutionary landscape of the most abundant CRISPR–Cas subtype in nature.

## Methods

### Cloning of Tma cas proteins

Three plasmids were used to overexpress *Tma* Cascade. The first (pCRISPR6) was generated by restriction enzyme cloning the Cas6 gene (GenBank ID: AGL50748.1) and a synthetic CRISPR array (Invitrogen Life Sciences) into pACYCDuet-1 (Novagen). The CRISPR array was designed to contain seven identical spacer sequences, corresponding to the third spacer of CRISPR locus eight (spacer 8.3) (Supplementary Table S1). The second plasmid (pCas758) was generated by first restriction enzyme cloning Cas7 (GenBank ID: AGL50735.1) into pHAT4 [57] with an N-terminal His6-SUMO tag. Cas5 (GenBank ID: AGL50734.1) and Cas8b1 (GenBank ID: AGL50736.1) were then added to this construct by Gibson assembly (NEB). The third plasmid (pCas11) was generated by ligation-independent cloning of Cas11 (Supplementary Table S1) in a pRSF-1b backbone (Novagen). Finally, pCas3 was generated by cloning Cas3 (GenBank ID: AGL50733.1) into pBAT4 with an N-terminal His6-SUMO tag. All genes, except Cas5, were amplified from genomic DNA isolated from *Tma* strain MSB8 (American Type Culture Collection). Cas5 was amplified from an *E. coli* codon-optimized gene synthesized by Invitrogen Life Technologies (Supplementary Table S1). Plasmid cloning and maintenance was performed in DH10B cells (Invitrogen). All enzymes were purchased from NEB. Oligos used for cloning are listed in Supplementary Table S2. All plasmids were verified by Sanger DNA sequencing.

### Expression and purification of the cas proteins

pCRISPR6, pCas758, and pCas11 were co-transformed into *E. coli* strain T7 Express (NEB). Cells were grown at 37°C in Lysogeny Broth supplemented with ampicillin, kanamycin, and chloramphenicol to an O.D. 600 between 0.3 and 0.4. At this point, protein expression was induced with 0.2 mM IPTG. Cells were harvested after ∼16 h. Pellets were resuspended in lysis buffer (500 mM KCl, 50 mM HEPES, pH 7.5, 10 mM imidazole, pH 7.5, 5.0% glycerol, and 1 mM TCEP) including protease inhibitors (PMSF, bestatin, E064, and pepstatin A) and lysed using an Emulsiflex-C5 homogenizer (Avestin). Lysate was then heat treated, with mixing, at 65°C for 10 min. Insoluble material was pelleted, and soluble lysate was collected and incubated with IMAC nickel affinity beads (Bio-Rad) for 30 min at room temperature with gentle agitation. Beads were then washed, and bound protein eluted with lysis buffer supplemented with 300 mM imidazole. The His6-SUMO tag was cleaved from Cas7b with SUMO protease. Uncleaved protein and SENP were removed by a second IMAC step. Finally, the cleaved protein was applied to a HiLoad 26/600 S200 Superdex column (Cytiva) equilibrated with running buffer (50 mM HEPES, pH 7.5, 200 mM KOAc, pH 7.5, and 1 mM TCEP). Cas3 was purified using the same protocol as Cascade, with the following modifications: expression was performed in *E. coli* strain BL21star (Thermo Fisher), and the lysis buffer contained 1.0 M NaCl instead of 0.5 M KCl. Protein purity was confirmed by SDS–PAGE. The integrity of the crRNA in the Cascade prep was confirmed by phenol chloroform extraction, followed by electrophoresis through urea–PAGE gels and SYBR Gold (Invitrogen) staining.

### Preparation of DNA targets

To generate pooled and single-target plasmids, dsDNA oligonucleotides (IDT) containing protospacer 8.3 flanked by NNN (Supplementary Table S2) were annealed and then restriction enzyme cloned into pHAT4 [57]. All enzymes were purchased from NEB. For single-target and library assays, targets (388 bp) were amplified using T7 promoter and terminator primers (Supplementary Tables S1 and S2). All targets were gel-purified before use. To facilitate imaging with single targets, the T7 promoter primer, which generates the target strand, was labeled with a Cy5 dye.

### DNA binding assays

Cascade DNA binding assays were performed in buffer containing 10 mM HEPES–KOH, pH 7.5, 200 mM potassium acetate, 10 mM magnesium acetate, 20 μM cobalt acetate, 2 mM ATP, 0.1 mg/ml bovine serum albumin, and 1 mM TCEP. For single-target binding, increasing concentrations of Cascade (0.2 to 6.4 nM in two-fold increments) were incubated at 80°C with 0.2 nM DNA for 30 min. Pooled library experiments were performed with 10 nM Cascade and 0.2 nM DNA library under the same conditions. Bound DNA was separated from free DNA by electrophoresis through 2.0% agarose gels. Single-target experiments were then imaged on a Typhoon imager (Cy5 channel). DNA ladders were imaged following ethidium bromide staining with an Odyssey imager. In pooled library experiments, bands corresponding to the bound and unbound fractions were gel purified (GeneJet Gel Extraction Kit, Thermo Fisher Scientific Inc.) before being subjected to NGS (see below).

### DNA cleavage assays

Reactions were performed in a buffer containing 10 mM HEPES–KOH, pH 7.5, 200 mM potassium acetate, 10 mM magnesium acetate, 20 μM cobalt acetate, 2 mM ATP, 0.1 mg/ml bovine serum albumin, 250 ng/μl salmon sperm DNA, and 1 mM TCEP. Cascade (10 nM) was incubated with a 4 nM PCR target at 80°C for 30 min. To start the reaction, Cas3 was added to the reaction to a final concentration of 250 nM. Single-target reactions were allowed to proceed, at 80°C, for 0, 0.5, 1, 2, 4, 8, and 32 min. Pool library reactions were allowed to proceed for 0 and 32 min under the same conditions. Reactions were terminated by the addition of EDTA to 50 mM and moving to ice. Zero time points were generated by adding the EDTA quencher before the addition of Cas3. Reactions were analyzed by electrophoresis through 2.0% agarose gels. Single-target reactions were imaged as above. In pooled library experiments, the bands corresponding to the uncleaved target, at times 0 and 32 min, were gel purified (GeneJet Gel Extraction Kit, Thermo Fisher Scientific Inc.) before being subjected to NGS (see below).

### Next-generation sequencing and analysis

Gel-purified DNA (corresponding to unbound and bound fractions in the target binding experiments and to the uncleaved fraction at time 0 and after 32 min in the cleavage assays) was amplified using primers containing partial Illumina adapters (Supplementary Table S2). Amplicons were gel purified (GeneJet Gel Extraction Kit, Thermo Fisher Scientific Inc.), adjusted to 20 ng/μl, and submitted for Illumina amplicon sequencing (Azenta Life Sciences, Amplicon-EZ service). In short, the Illumina MiSeq platform, or equivalent, was used to collect paired-end 2 × 250 bp reads. All experiments were independently performed four or five times and sequenced in separate Amplicon-EZ runs.

Paired-end reads were merged with PANDAseq [58]. To determine read counts for all possible PAMs in each sample, the resulting files were searched for the 64 possible PAM-protospacer sequences. The counts for individual sequences were normalized by dividing by the total number of reads in the sample. To calculate binding enrichment, the normalized read counts in the bound fraction were divided by the normalized read counts in the corresponding unbound fraction. To calculate depletion in cleavage experiments, the normalized read counts at time 0 were divided by the normalized read counts at 32 min. Finally, enrichment and depletion scores for each replicate were averaged and standard deviations determined (Supplementary Table S3).

## Supplementary Material

Supplementary Figure S1-S3 and Tables S1-S2

Supplementary S3

## Data Availability

Next-generation sequencing data have been deposited at GEO under accession number GSE320408 [61]. All other data are included within the main article and its supplemental files. Experimental materials are available from the corresponding author on request.

## Open Access

Open access for this article was enabled by the participation of Johns Hopkins University in an all-inclusive Read & Publish agreement with Portland Press and the Biochemical Society.

## Abbreviations

Cas: CRISPR-associated
CRISPR: clustered regularly interspaced short palindromic repeats
crRNA: CRISPR-RNA
PAM: protospacer adjacent motif
Tma: *Thermotoga maritima*
NGS: Next Generation Sequencing
