# Supplementary material for: Target discrimination and PAM profiling of the Thermotoga maritima type I-B CRISPR system
Source: Biochem J. 2026 May 21;483(6):981–92. doi: 10.1042/BCJ20260189 (PMC13199841; doi:10.1042/BCJ20260189)
Supplement: Supplementary Figure S1-S3 and Tables S1-S2 [file BCJ-2026-0189_supp.pdf]

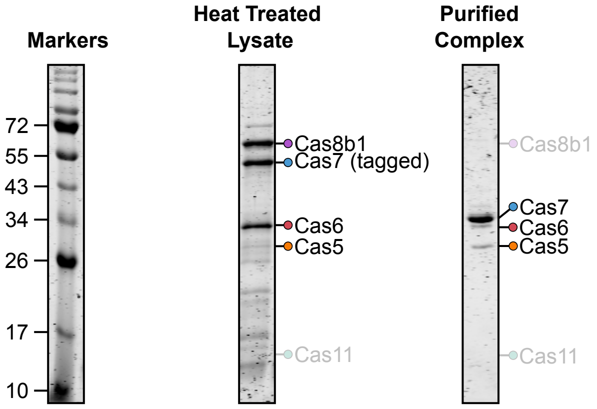

Figure S1: Purification of Tma Cascade without standalone Cas11

SDS-PAGE of purified Cascade subcomplex that lacks Cas8b1 and Cas11 subunits. The expected position of Cascade subunits is indicated.

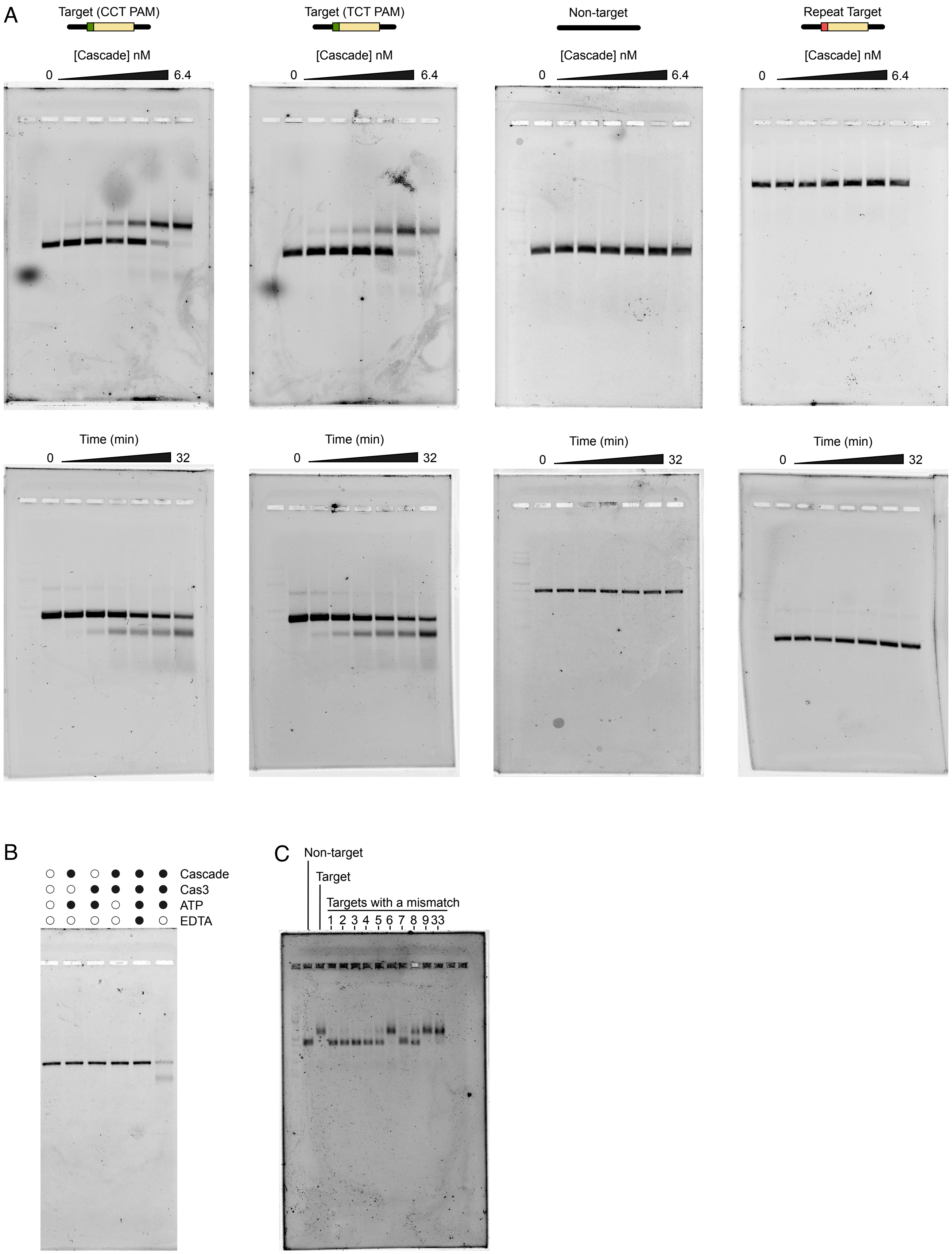

Figure S2: Full gels from the reconstituted activity of the *Tma* type I-B CRISPR system
(A) Full gels corresponding to Figure 2B binding (top) and DNA cleavage (bottom).
(B) Full gel from Figure 2D. (C) Full gel from Figure 2E.

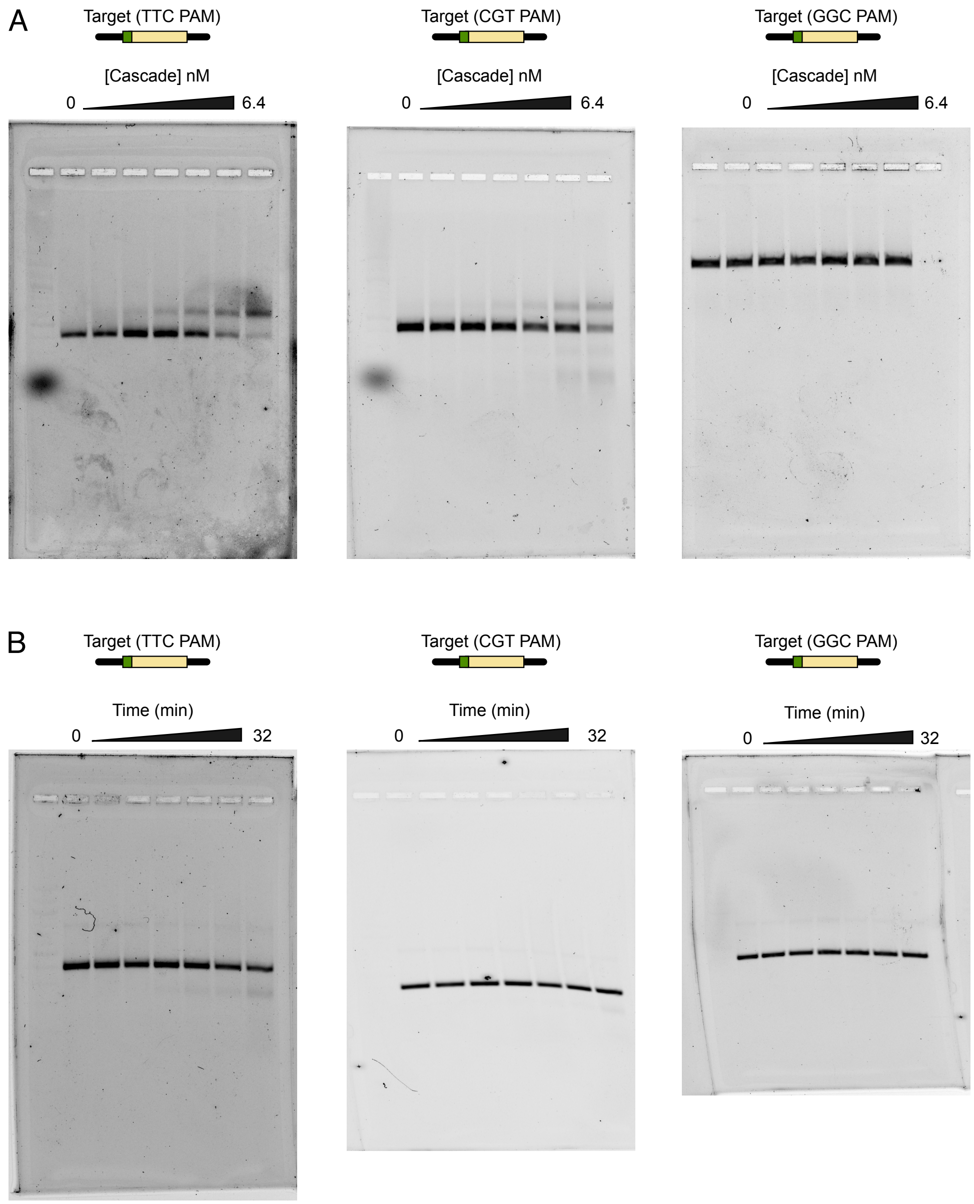

Figure S3: Effect of PAM sequence on the *Tma* Type I system activity.
(A) Full gels from Figure 3D - DNA binding assays.
(B) Full gels from Figure 4C - DNA cleavage assays.

Table S1. Sequences of DNA and RNA used in this study

Name	Sequence (5' to 3')
CRISPR array	GAAGGCCGTCAAGGCCGCATAGATCTGTTTCAATAATTCCTTAGAGGTATGGAA<br>ACCCGTCCCTTCTTTCCTGTCTTTTTGCCTCCTTTAAGTGTTTCAATAATTCCT<br>TAGAGGTATGGAAACCCGTCCCTTCTTTCCTGTCTTTTTGCCTCCTTTAAGTGT<br>TTCAATAATTCCTTAGAGGTATGGAAACCCGTCCCTTCTTTCCTGTCTTTTTGC<br>CTCCTTTAAGTGTTTCAATAATTCCTTAGAGGTATGGAAACCCGTCCCTTCTTT<br>CCTGTCTTTTTGCCTCCTTTAAGTGTTTCAATAATTCCTTAGAGGTATGGAAAC<br>CCGTCCCTTCTTTCCTGTCTTTTTGCCTCCTTTAAGTGTTTCAATAATTCCTTA<br>GAGGTATGGAAACCCGTCCCTTCTTTCCTGTCTTTTTGCCTCCTTTAAGTGTTT<br>CAATAATTCCTTAGAGGTATGGAAACCCGTCCCTTCTTTCCTGTCTTTTTGCCT<br>CCTTTAAGTGTTTCAATAATTCCTTAGAGGTATGGAAACCTCGAGCTGGGCCTC<br>ATGGGCCTTCC
*TmaCas5 **	ATGAAAGTGCTGGTTTTTGATGTTAGCGCACCGTATGCACTGTTTCGTCGTCCG<br>TATACCACCACCAGTAGCTATACCCTGCCGTTTCCGCCTCGTACCACACTGCTG<br>GGTTTAGTTGGTTGTGTTCTGGGTTATAGCACACCGGAACGTCTGGATAGCGCA<br>AAAGTTGCAGTTCAGATTAAAAACCCGCTGAAATTTCTGCGTACCGGCACCAAT<br>TTTGTGGAAACCAAAAAAGATAAAAAGGCCAGCAAACGTACCCGTATTAGCCTG<br>CAGCTGCTGAAAAATCCGGCATATCGTGTGTTTTTTAGCTGGGAAGATGAAGAT<br>TTTGAGCGCCTGAAAAACCTGCTGGAACATAGCGAAACCATTTTTACCCCGTAT<br>CTGGGTGTTGCAAGCTTTATTGCACGTCTGAATTATGTGGGTAAATATGAAGCA<br>ACCCGTGTTGCAGATTTTCCGTGTGAAGTTCATACCGTTGTTCCGAATACCGTT<br>AAACTGCTGCCGGAACCGAGCCATTATCTGATTTTTGAACGTGTTACCCGCAAA<br>ATGGATAAAGAACGTAATATGCTGGAAAGCGCAGTGTATATCTTTAAACGTGAT<br>CTGAGTCCGGTTAAAGTTGAAGGTGGTGAAGTTTGGCGTGTTGGTGAACAGAAT<br>ATTGTGTGGATGTGA
TmaCas11	ATGAAGGGTGAAGATTTCTTCTCCAGGTATCCGGAGTTTTTCGATGAACCGTGG<br>AAGAAAGCAGTCTTTTTAGAAGGAGTTCTGGCAAATTACCTTCTTTATCTGCAG<br>TACGTTAAAAGAAACTCGAAGGCTTTCACGAAGAAACTGAAAGGACTCAGACTG<br>ACAAAGAGAGATGTGGAGGGGCTTCTTCCGGAAATCAGAGCCAAGATCGAAGCC<br>TACGGTGGAATGAGTGAAAGTGTGGCAGAGCTTTTCAGGGAGACTGCTGAAGCT<br>TTCCTCGAAGCTGGGAACTGGTCAGCATCACCCGACGAGATCAGTTTTGTCTTC<br>GTCTCGGGCTTTCTCTCGGGAAAACTTTCTTCAGGGAGGTAGGGGTCGATGAAT<br>CCAGTGAAGAACAGGAGTGA
*PCR Target ***	TAATACGACTCACTATAGGGAATTGTGAGCGGATAACAATTCCTCTAGAAATAA<br>TTTTGTTTAACTTTAAGAAGGAGATATATCCATGAACACCATTCATCACCATCA<br>CCATCACAACACTAGTGGATCTGGTGGTGGCGGCCGCCTGGTTCCGAGGGG<br>ATCCATGTCTGAAAACCTGTACTTCCAGGGTTCCATGACTTAAAGGAGGCAAAA<br>AGACAGGAAAGAAGGGACGG**NNN**TCGATCGAGGGATCCGGGCCCTCTAGATGCG<br>GCCGCATGCATAAGCTTGAGTATTCTATAGTGTCACCTAAATCCCAGCTTGATC<br>CGGCTGCTAACAAAGCCCGAAAGGAAGCTGAGTTGGCTGCTGCCACCGCTGAGC<br>AATAACTAGC

* *Codon Optimized*
*** N = A, T, G or C*

Table S2. Sequences of synthetic DNA oligonucleotides used in this study

Name	Sequence (5' to 3')
TmaCas6.F	GTGTGTCCATGGGTAGATTGAAAGTTTCTTTTCAGG
TmaCas6.R	GTGTGTGCGGCCGCTCACCTCTCATATTTTTTCAAGGC
CRISPR.F	GAAGGCCGTCAAGGCCGCAT
CRISPR.R	GGAAGGCCCATGAGGCCCAG
TmaCas7.F	GTGTGTACCGGTGGAATGAATCCAGTGAAGAACAGGAG
TmaCas7.R	GTGTGTCTCGAGTCACCACTCCAGTTTTACAAG
TmaCas5.F	GCTTGTAAAACTGGAGTGGTGAGCGGCCGCAAGGAGATATACCATGAAAGTGCTGGTTTTTGATG
TmaCas5.R	GTTCTGCTCTCCCACCCTCCATGGTATATCTCCTTGCGGCCGCTTATCACATCCACACAATATTC
TmaCas8b1.F	GAATATTGTGTGGATGTGATAAGCGGCCGCAAGGAGATATACCATGCTTGAAAAGATATACAATCTTG
TmaCas8b1.R	CTCAAGCTTATGCATGCGGCCGCTCACTCCTGTTCTTCACTGGATTC
TmaCas11.F	TTTAAGAAGGAGATATAGATCATGAAGGGTGAAGATTTCTTCTCC
TmaCas11.R	TTATGGAGTTGGGATCTTATTACTCCTGTTCTTCACTGGATTCA
TmaCas3.F	GTGTGTACCGGTGGAATGGAACCTTTTAAATATATATG
TmaCas3.R	GTGTGTGCGGCCGCTTATTTGGGATTTGCAGGGATG
Target.F *	CATGACTTAAAGGAGGCAAAAAGACAGGAAAGAAGGGACGG**NNN**
Target.R *	TCGA**NNN**CCGTCCCTTCTTTCCTGTCTTTTTGCCTCCTTTAAGT
T7.F	TAATACGACTCACTATA
T7.R	GCTAGTTATTGCTCAGCGG
T7.Illumina.F	ACACTCTTTCCCTACACGACGCTCTTCCGATCTTAATACGACTCACTATA
T7.Illumina.R	GACTGGAGTTCAGACGTGTGCTCTTCCGATCTGCTAGTTATTGCTCAGCGG

** N = A, T, C or G*
